# Synucleinopathy-associated pathogenesis in Parkinson’s disease and the potential for brain-derived neurotrophic factor

**DOI:** 10.1038/s41531-021-00179-6

**Published:** 2021-04-12

**Authors:** Kathryn M. Miller, Natosha M. Mercado, Caryl E. Sortwell

**Affiliations:** 1 Department of Translational Neuroscience, College of Human Medicine, Michigan State University, Grand Rapids, MI USA; 2 Neuroscience Graduate Program, College of Natural Science, Michigan State University, East Lansing, MI USA; 3 Hauenstein Neuroscience Center, Mercy Health Saint Mary’s, Grand Rapids, MI USA

**Keywords:** Parkinson's disease, Cell biology

## Abstract

The lack of disease-modifying treatments for Parkinson’s disease (PD) is in part due to an incomplete understanding of the disease’s etiology. Alpha-synuclein (α-syn) has become a point of focus in PD due to its connection to both familial and idiopathic cases—specifically its localization to Lewy bodies (LBs), a pathological hallmark of PD. Within this review, we will present a comprehensive overview of the data linking synuclein-associated Lewy pathology with intracellular dysfunction. We first present the alterations in neuronal proteins and transcriptome associated with LBs in postmortem human PD tissue. We next compare these findings to those associated with LB-like inclusions initiated by in vitro exposure to α-syn preformed fibrils (PFFs) and highlight the profound and relatively unique reduction of brain-derived neurotrophic factor (BDNF) in this model. Finally, we discuss the multitude of ways in which BDNF offers the potential to exert disease-modifying effects on the basal ganglia. What remains unknown is the potential for BDNF to mitigate inclusion-associated dysfunction within the context of synucleinopathy. Collectively, this review reiterates the merit of using the PFF model as a tool to understand the physiological changes associated with LBs, while highlighting the neuroprotective potential of harnessing endogenous BDNF.

## Introduction

Parkinson’s disease (PD) affects over a million Americans and results in nearly $25 billion per year in health care costs as well as immeasurable personal costs to patients and families. It is now appreciated that PD is a complex, multifaceted disorder that impacts both the central and peripheral nervous systems with patients experiencing symptoms ranging from motor dysfunction to constipation to dementia; all contributing to a significant detriment in quality of life. However, the cardinal motor symptoms of tremor, rigidity, akinesia/bradykinesia and postural instability first described by James Parkinson in 1817 are still requisite for diagnosis and are the primary target for therapeutic intervention^1^. PD motor symptoms are caused by the loss of dopaminergic transmission in the striatum due to progressive loss of dopamine neurons in the substantia nigra pars compacta (SNpc) and their projections to the caudate and putamen. As a result, current pharmacotherapies attempt to augment nigrostriatal dopamine transmission. Unfortunately, these approaches are not disease-modifying, with pharmacotherapy ultimately losing therapeutic efficacy and causing troublesome side effects as the disease progresses.

The lack of disease-modifying treatments for PD is partly due to an incomplete understanding of the disease’s etiology. PD belongs to a family of disorders termed synucleinopathies, which are characterized pathologically by the deposition of the protein alpha-synuclein (α-syn) into neuronal inclusions termed Lewy bodies (LBs). Despite the identification of genetic forms of PD (reviewed in ref. ^2^), the molecular etiology underlying disease origin and progression remain unknown. Nevertheless, abnormal α-syn proteostasis is a common factor between both sporadic and familial forms of PD (reviewed in ref. ^3^). Together with the loss of nigrostriatal dopamine neurons, accumulation of α-syn into LBs is the pathological hallmark of PD. There are many ideas surrounding the mechanism by which aberrant α-syn proteostasis may contribute to PD (reviewed in ref. ^4^); however, each is contested, and none have been proven outright. Further, whether LBs themselves directly cause toxicity or are merely a cellular marker associated with pathogenic processes has yet to be determined. In either case, understanding the pathogenic mechanisms associated with, or induced by, LB formation is critical to the development of disease-modifying treatments.

## α-synuclein

α-syn is a small (14 kDa) protein encoded by the SNCA gene. It is abundantly expressed in the nervous system^5^ and exists in a natively unfolded state under normal physiological conditions, though its conformation changes depending on its environment^6^ and interactions with binding partners^7^. For example, α-syn has a strong affinity for high curvature lipid membranes (i.e. vesicles), and it changes conformation from two alpha helices^7^ to one alpha helix upon interacting with them^8–10^. Functionally, α-syn is enriched in synaptic terminals where it is known to play critical roles in neurotransmission^3,5,11^. It is thought to mediate trafficking, docking, and endocytosis of synaptic vesicles via interactions with soluble NSF attachment protein receptor (SNARE) complex proteins^11,12^ and synaptic vesicles directly^13^. Of relevance to nigrostriatal neurons, α-syn is involved in dopamine synthesis^14,15^, handling^16,17^, and release^11,18,19^, and is posited to serve as a negative regulator of synaptic transmission^3^.

α-syn remains an ‘intrinsically disordered’ protein due to its dynamic nature with no clear tertiary structure, making it particularly vulnerable to aggregation^6,20^. Many conditions can promote this transition from soluble, functional α-syn into insoluble fibrils (reviewed in^3^), and once this process begins, it proceeds in a feed-forward manner in which oligomers form and become fibrils which seed and recruit soluble α-syn into more fibrils, a process that once escalated is largely irreversible^21^. While α-syn oligomers and aggregates have not been proven to be directly toxic, they are consistently associated with toxicity (reviewed in refs. ^22,23^). Within PD and other synucleinopathies, α-syn transforms from a soluble, functional protein to a phosphorylated, aggregated, protein (i.e., LBs) that becomes associated with pathogenic consequences^24^.

## Insights derived from postmortem PD tissue

Three different technical approaches that provide insight into the pathophysiological mechanisms associated with LBs have been employed in studies examining PD pathogenesis in human postmortem tissue (Table 1). First, quantitative immunofluorescent techniques have been used to examine proteins within LB-containing vs. non-LB-containing neurons. These studies have demonstrated that LB-containing neurons exhibit reduced ubiquitin-proteasome system (UPS) and lysosomal markers^25^, kinesis motor proteins, and pro-survival myocyte enhancer factor 2D^26,27^, and increased DNA strand breaks^28^ and toll-like receptor 2^29^. Whereas this immunofluorescence approach maintains the specificity of the LB vs. no LB comparison, it is limited by the number of different proteins that can be analyzed at any one time.

**Table 1:** Synucleinopathy-associated pathogenesis in postmortem PD tissue.

Authors	Year	Comparator	Findings
QUANTITATIVE IMMUNOFLUORESCENCE			
Chu et al.^25^	2009	LB vs non-LB neurons	Decreased ubiquitin-proteasome system and lysosomal markers with LBs
Chu et al.^26^	2011	LB vs non-LB neurons	Decreased myocyte enhancer factor 2D with LBs
Chu et al.^27^	2012	LB vs non-LB neurons	Decreased kinesis motor proteins in neurons with LBs Increased expression of dynein in neurons with LBs
Dzamko et al.^29^	2017	LB vs non-LB neurons	Increased toll-like receptor 2 in neurons with LBs
Schaser et al.^28^	2019	LB vs non-LB neurons	Increased DNA double stranded breaks in neurons with LBs
WHOLE TISSUE MICROARRAY			
Grunblatt et al.^30^	2004	PD SN vs control SN	68 downregulated in PD involved in signal transduction, protein degradation, dopamine handling, ion transport, and energy pathways. 69 upregulated in PD involved in protein modification, metabolism, transcription, and inflammation.
Hauser et al.^31^	2005	PD SN vs control SN	96 genes differentially expressed. Main pathways were chaperones, ubiquitination, vesicle trafficking, and mitochondrial function
Duke et al.^32^	2006	PD SN vs control SN	Downregulation of pathways related to ubiquitin-proteasome system and mitochondrial function.
Elstner et al.^33^	2009	PD SN vs control SN	4 genes differentially expressed. Pathways were mitochondrial function, dopamine metabolism, axon guidance, and vesicle transport.
Botta-Orfila et al.^34^	2012	PD LC vs control LC	Differential expression of genes related to synaptic transmission, neuron projection, and immune system related pathways
Dijkstra et al.^35^	2015	PD SN vs ILBD SN vs control SN	Dysregulation of pathways related to axonal guidance, endocytosis and immune response (ILBD) as well as dysregulated mTOR and EIF2 signaling in both ILBD and PD.
LASER CAPTURE MICRODISSECTION			
Lu et al.^36^	2005	PD SN neurons with LBs vs PD SN neurons without LBs	Increased USP8 (pro-UPS function) Increased ANP32B (proapoptic) Decreased KLHL1 and BPAG1 (cytoskeleton organization), Decreased Stch (encodes HSP 70)
Cantuti-Castelvetri et al.^37^	2007	PD SN neurons vs control SN neurons Both male and female	Females: Alterations in genes with protein kinase activity, genes involved in proteolysis and WNT signaling pathway. Males: Alterations in protein-binding proteins and copper-binding proteins.
Elstner et al.^38^	2011	PD SN neurons vs control SN neurons	Downregulation of genes coding for mitochondrial and ubiquitin-proteasome system proteins
Grundemann et al.^145^	2011	PD SN neurons vs control SN neurons	Increased SNCA expression
Lin et al.^39^	2012	ILBD SN neurons vs PD SN neurons vs control SN neurons	Increased mitochondrial DNA mutations in early PD/ILBD group compared to late stage and controls
Grunewald et al.^40^	2016	PD SN neurons vs control SN neurons	Reduced respiratory chain complex I and II
Su et al.^42^	2017	PD SN neurons vs control SN neurons	Decreased SNCA expression, no changes in Nurr1, RET, PARK7, SLC18A2, BDNF, DDC, TH, MEF2D or PITX3
Duda et al.^41^	2018	PD SN neurons vs control SN neurons	Dysregulation in genes encoding for ion channels, dopamine metabolism proteins, and PARK.

*LB* Lewy body, *PD* Parkinson’s disease, *SN* substantia nigra, *LC* locus coeruleus, *ILBD* incidental Lewy body disease.

In contrast to the immunofluorescence approach, several studies have used the approach of microarray profiling (Table 1) to compare whole nigral tissue from varying stages of PD to control brains, identifying a wide array of dysregulated genes involved in synaptic transmission, protein degradation, dopamine handling, ion transport, transcription, inflammation, vesicle trafficking, axon guidance and mitochondrial function^30–35^. The whole tissue microarray approach has the advantage of an unbiased survey of gene expression changes, but at the cost of losing the specificity necessary to precisely identify the transcriptome of neurons possessing LBs. This is due to the fact that LB and non-LB-containing neurons (and other cell types) are present within the whole tissue punch. Further, depending on disease stage, the whole tissue approach can be confounded by the loss of nigral neurons themselves.

The approach of laser capture microdissection (LCM) combined with gene expression analysis has been used to compare dopaminergic nigral neurons in PD vs control brains, allowing for single neuron resolution to be combined with either focused or unbiased expression analysis (Table 1). To the best of our knowledge, only one LCM study has specifically compared expression differences between LB-containing and non-LB-containing nigral neurons^36^. This focused study, conducted in a small sample size, suggested that LB-containing nigral neurons have increased expression of proapoptotic and pro-UPS genes, and decreased expression of genes associated with cytoskeletal organization and molecular chaperones. The remainder of LCM studies have examined transcript differences between nigral dopamine neurons in disease and healthy control nigral dopamine neurons, without using the presence of LBs as a determining selection factor. These studies show that nigral dopamine neurons from PD brains have alterations in genes associated with protein kinase activity, UPS functioning, mitochondrial function, dopamine metabolism, and ion channels^37–41^. Less agreement has surfaced from LCM studies with regards to the expression of α-syn itself, with earlier studies suggesting increased SNCA expression in PD nigral neurons [80], and a more recent analysis suggesting no change in SNCA expression or trophic factor signaling and dopamine metabolism genes [81].

**Fig. 1:**
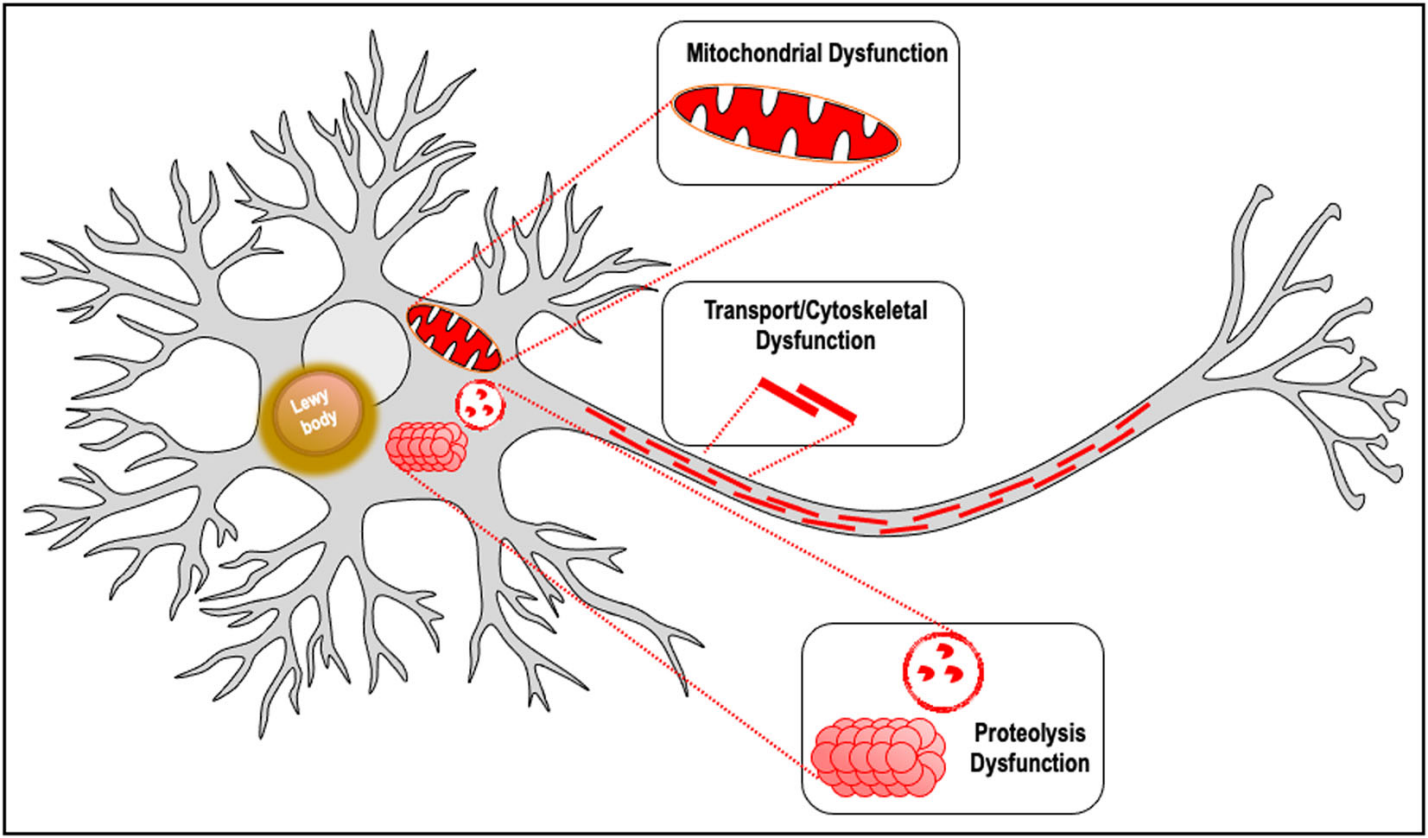
Pathogenic mechanisms consistently associated with Lewy bodies across multiple approaches. Both immunofluorescence and LCM of LB-containing nigral neurons reveal alterations in proteolysis markers^25,36^ as well as alterations in transport/cytoskeleton organization^27,36^. Some whole SN tissue analysis and LCM studies of nigral DA neurons also have detected proteolysis and transport/cytoskeletal dysfunction^30–32,37,38^. Mitochondrial dysfunction is quite frequently detected by both whole SN tissue analysis and LCM approaches^30–32,38,39,145^ however the association specifically with LBs has not directly been established.

In order to understand what pathogenic mechanisms are consistently associated with LBs, it is reasonable to look for consensus across different methodological approaches with an emphasis on LB-specific analyses (Fig. 1). Both immunofluorescence and LCM of LB-containing nigral neurons reveal alterations in proteolysis markers^25,36^ as well as alterations in transport/cytoskeleton organization^27,36^. Some whole SN tissue analysis and LCM studies of nigral dopamine neurons also have detected proteolysis and transport/cytoskeletal dysfunction^30–32,37,38^. Mitochondrial dysfunction is quite frequently detected by both whole SN tissue analysis and LCM approaches^30–32,38–40,42^; however, the association specifically with LBs has not directly been established. Despite these efforts using PD brain tissue, the pivotal pathogenic mechanisms associated with the formation of LBs have yet to be revealed. The heterogeneity of PD combined with the difficulty of gleaning mechanistic insight using analysis of static postmortem tissue further confound the potential for our understanding of LB-associated pathogenesis. Fortunately, an alternative approach is providing new information of the dynamic cellular alterations associated with the formation of α-syn inclusions.

## Insights from the α-syn PFF model

Since the earliest observation of LBs, the question of what role this intracellular structure plays in degeneration remains unanswered. LBs may trigger cytotoxic events, be beneficial, or may simply represent an artifact that is inconsequential to either pathogenesis or neuroprotection. Transgenic animal models in which α-syn aggregates are formed rarely lead to overt degeneration^43–46^, limiting their utility for understanding the relationship between α-syn aggregation and degeneration. However, a relatively recent model described in wildtype mice^47^ demonstrated the ability of intrastriatal injection of preformed α-syn fibrils (PFFs) to seed LB-like aggregates in the SN and multiple cortical regions. PFFs are taken up into neurons^48^ and once inside initiate a conversion of normal α-syn into phosphorylated and misfolded α-syn, ultimately accumulating to form LB-like aggregates^48,49^. Importantly, a definitive link between PFF-seeded pathological α-syn aggregation and eventual neuronal death has been established in this model^50^.

Although multiple studies have examined the degenerative phenotype induced by PFF injections to mice and rats^47,51–53^, studies using PFFs in primary neuronal cultures prove particularly useful in revealing the intracellular events observed in tandem with the formation and maturation of PFF-triggered LB-like inclusions. Neurons in which phosphorylated α-syn inclusions form following α-syn PFF exposure exhibit multiple structural, protein, and transcriptomic changes that are associated with pathophysiological processes and, in the case of longer in vitro intervals, cell death (Table 2). Specifically, PFF-initiated α-syn inclusions result in decreased expression of synaptic proteins^48,54,55^, impairments in axonal transport^49^, and mitochondrial impairment^54,56,57^. Structurally and functionally, α-syn inclusion-bearing neurons display impaired excitability and decreased spine density^48,58,59^. Notably, results from the in vitro PFF model reveal heavy overlap with results from LB-containing neurons from PD brains, particularly with regard to transport/cytoskeleton disorganization and mitochondrial dysfunction. The most comprehensive study to date in cultured neurons with PFF-seeded α-syn inclusions was conducted by Mahul-Mellier and colleagues^55^ and examined longitudinal transcriptomic alterations in neurons as α-syn inclusions matured. In addition to observing the decreased expression of synaptic genes, cytoskeletal organization genes, and mitochondrial genes, this study also revealed decreased expression of a gene that has previously received little attention with regard to α-syn inclusion-associated alterations: brain-derived neurotrophic factor (BDNF). Specifically, out of the 11767 total mouse genes that were examined, only 0.05% were significantly decreased across all time points, including *bdnf*. In inclusion-bearing neurons, *bdnf* decreased 1.2 fold at day 7, 2.32 fold at day 14, and 2.5 fold at day 21. Further, of all the genes significantly decreased at day 21, the magnitude of *bdnf* decrease was greater than 98% of all the others. In other words, only 9 other genes (out of 11767 total, 562 were downregulated) exhibited a greater magnitude of reduction than *bdnf* at day 21. The 9 other downregulated genes included genes encoding proteins with functions related to protein coding and processing (Spink8, Fam150b, Pcsk1), transcription (Npas4), G protein signaling (Rgs4), adhesion (Bves), lipoprotein metabolism (Lipg), vesicle trafficking (Sv2b), and the gene for the tachykinin receptor 3 (Tacr3).

**Table 2:** Synucleinopathy-associated pathogenesis in PFF culture studies.

Authors	Year	Comparator	Findings	Culture Age	PFF Species
Volpicelli-Daley et al.^48^	2011	Hippocampal neurons with and without α-syn aggregates	Decreased expression of multiple synaptic proteins. Impairments in neuronal excitability and connectivity.	E16-E18 mouse	Mouse
Volpicelli-Daley et al.^49^	2014	Hippocampal neurons with and without α-syn aggregates	Impairment of axonal transport of RAB7 and TrkB- containing endosomes and autophagosomes. Accumulation of pERK5.	E16-E18 mouse	Mouse
Tapias et al.^54^	2017	Mesencephalic dopamine neurons with and without α-syn aggregates	Decreased expression of synaptic proteins. Alterations in axonal transport-related proteins. Impaired mitochondria. Increased oxidative stress.	E17 rat	Human
Froula et al.^58^	2018	Hippocampal neurons with and without α-syn aggregates	Decreased mushroom spine density. Increased excitatory postsynaptic currents. Increased presynaptic docked vesicles. Decreased frequency and amplitude of spontaneous calcium transients.	E16-E18 mouse	Mouse
Grassi et al.^56^	2018	Hippocampal neurons with and without α-syn aggregates	pSyn* induces mitochondrial toxicity and fission, energetic stress and mitophagy.	E16-E18 mouse	Mouse
Wu et al.^59^	2019	Hippocampal neurons with and without α-syn aggregates	Decreased excitatory postsynaptic current frequency. Altered dendritic spines.	E16-E18 mouse	Human
Wang et al.^57^	2019	Cortical neurons with and without α-syn aggregates	Deficits in mitochondrial respiration	P1 mouse and rat	Mouse
Mahul-Mellier et al.^55^	2020	Hippocampal neurons with and without α-syn aggregates	Transcriptomic changes over time: D7: 75 total genes (27 upregulated, 48 downregulated) encoding for proteins located within synapses, axons, or secretory and exocytic vesicles. Genes encoding for proteins involved in neurogenesis and the organization, growth, and the extension of the axons and dendrites D14: 329 total genes (106 upregulated, 223 downregulated) linked to the synaptic, neuritic, and vesicular cellular compartments. Genes associated with neurogenesis, calcium homeostasis, synaptic homeostasis, cytoskeleton organization, response to stress, and neuronal cell death process. D21: 1017 total genes (455 upregulated, 562 downregulated) with enrichment in genes encoding for proteins related to the ion channel complex, plasma membrane protein complex, cell–cell junctions, synaptic functions, response to oxidative stress and mitochondria. D14 vs. D21: Differential expression of genes associated with mitochondrial and synaptic functions.	P0 mouse	Mouse

*D* day in vitro. *Culture age* animal age animals at the time of primary neurons harvest.

## Brain-derived neurotrophic factor

The best-studied neurotrophic factors in the context of PD are glial cell line-derived neurotrophic factor (GDNF), neurturin (NTN), BDNF, cerebral dopamine neurotrophic factor (CDNF), and mesencephalic astrocyte-derived neurotrophic factor (MANF)^60^. The potential for trophic factors to protect nigrostriatal neurons in PD has been extensively explored in recent years, with the GDNF family ligands, GDNF and NTN, advancing to clinical trials that have all ultimately failed to provide significant clinical improvement in PD subjects^61–66^. To date, clinical research efforts investigating the neuroprotective potential of trophic factors in PD have been mainly directed toward GDNF and NTN. As a result, the neuroprotective potential of other trophic factors has been explored only sparingly. Of these, BDNF is a member of the mammalian neurotrophin family and is abundantly expressed in the central nervous system from development through adulthood, where it plays critical roles in neuronal survival, migration, axonal and dendritic outgrowth, synaptogenesis, synaptic transmission, and synaptic plasticity^67–72^. BDNF also promotes neuroprotection after injury by inhibiting pro-apoptotic molecules^73^.

Examination of BDNF mRNA and protein levels in PD subjects has revealed alterations relative to aged matched controls. Postmortem examination suggests that BDNF mRNA and protein are downregulated in the SNpc of patients with PD^74–76^. This decrease in nigral BDNF mRNA correlates with both decreased soma size and neuron survival, suggesting that individual nigral neurons with low BDNF levels may be particularly vulnerable to degeneration^74^.

The biological activity of BDNF is tightly regulated by its gene expression, axonal transport, and release. It is well-known that BDNF is synthesized and released in an activity-dependent manner and as such, endogenous extracellular BDNF levels are extremely low^72^. BDNF release is dependent on stimulus pattern, with high-frequency bursts being the most effective^77^. The *bdnf* gene has nine promoters that produce 24 different transcripts, all of which are translated into a single, identical, mature dimeric protein^78^. This allows for tight, activity-dependent regulation whereby specific exon-containing transcripts are differentially regulated by specific neuronal activities including physical exercise, seizures, antidepressant treatment, and regular neuronal activation^79–85^. Neuronal activity also regulates the transport of BDNF mRNA into dendrites allowing for locally translated BDNF to modulate synaptic transmission and synaptogenesis^86–88^.

BDNF protein is initially synthesized as a precursor protein (preproBDNF) in the endoplasmic reticulum. Following cleavage of the signal peptide into a 32-kDa proBDNF protein, it is either cleaved intracellularly into mature BDNF (mBDNF) or transported to the Golgi for sorting into either constitutive or regulated secretory vesicles for release (reviewed in ref. ^89^). Mature BDNF is the prominent isoform in the adult, whereas proBDNF is highly expressed at early postnatal stages^90^. proBDNF was initially thought to be an inactive, intracellular precursor for mBDNF in the adult, but it is now understood to be a secreted, biologically active molecule with pro-apoptotic effects^90–94^. While proBDNF regulation and secretion are still relatively unclear processes, both proBDNF and mBDNF are packaged into vesicles of the activity-regulated secretory pathway with the secretion of proBDNF more prominent than mBDNF^91^.

BDNF protein is found widespread throughout the CNS both pre- and postsynaptically and affects neuronal survival, growth/arborization, and synaptic plasticity^95^. It undergoes both retrograde and anterograde transport^96,97^; therefore the site of BDNF synthesis and function are not always the same. For example, BDNF protein is abundant in the striatum where it is critical for normal function. However, there is relatively little BDNF mRNA in the striatum^98^. Instead, the overwhelming majority of striatal BDNF is anterogradely transported from the cortex and to a lesser extent from the SNpc^97,99^. BDNF release is triggered in an activity-regulated, Ca^2+^-dependent manner. This can occur by presynaptic influx of Ca^2+^^100^, postsynaptic influx of Ca^2+,^^101^, or from release of intracellular Ca^2+^ stores^102^.

BDNF binds and activates two known surface receptors: mBDNF binds to tropomyosin related-kinase receptor B (TrkB) whereas proBDNF binds to pan neurotrophin receptor (p75NTR; p75)^103^. TrkB is part of the tyrosine kinase family of receptors, along with TrkA and TrkC. BDNF binds to TrkB with high affinity inducing a pro-survival cascade. In contrast, all neurotrophins bind to p75 with low affinity (also known as low-affinity nerve growth factor receptor, LNGFR) inducing apoptosis, and the balance of p75 and the Trk receptors ultimately determines cell survival or death (reviewed in^104^). The majority of BDNF signaling is attributed to mBDNF binding and activating TrkB. However, evidence suggests that proBDNF binds p75, and pro- and mBDNF elicit opposing synaptic effects through activation of their respective receptors^90–93,105^. Moreover, mBDNF also binds a truncated TrkB receptor lacking the tyrosine kinase domain involved in downstream signaling^106,107^. Thus, when bound to p75 or truncated TrkB, BDNF is functionally inhibited from activating the canonical BDNF-TrkB signaling pathway, acting as a dominant-negative regulatory mechanism^108^.

BDNF-TrkB signaling can activate two distinct postsynaptic signaling pathways: the canonical and the noncanonical pathways. In the canonical pathway, three signaling cascades have been identified: (1) the mitogen-activated protein kinase/extracellular signal related-kinase (MAPK/ERK) cascade, (2) the phosphatidylinositol 3-kinase/AKT (PI3K/AKT) cascade, and (3) the phospholipase C gamma (PLCγ) cascade^109,110^. MAPK/ERK and PI3K/AKT cascades mediate translation and trafficking of proteins^95^, whereas PLCγ mediates transcription via intracellular Ca2+ regulation and cyclic adenosine monophosphate and protein kinase activation^109^. Collectively, these cascades affect neuronal survival, growth/ arborization, and synaptic plasticity^95^. In the noncanonical pathway, intracellular PI3K-Akt signaling results in phosphorylation of the NMDA receptor 2B subunit,^111–113^, resulting in potentiated responses. BDNF noncanonical signaling has also been suggested to have effects on presynaptic dopamine release and reuptake^114^. These immediate phosphorylation events in the noncanonical pathway occur at a much faster rate than the translational and transcriptional events in the canonical pathway. Thus, BDNF can exert a multitude of effects on the basal ganglia over various time spans.

## Can BDNF counteract synucleinopathy-associated pathogenesis?

α-syn inclusions seeded by PFFs are associated with an early and profound decrease in BDNF mRNA in cultured neurons^55^. Similarly, BDNF mRNA and protein are downregulated in the SNpc of patients with PD^74,75^. This decrease in nigral BDNF mRNA correlates with both decreased soma size and neuron survival, suggesting that individual nigral neurons with low BDNF levels may be particularly vulnerable to degeneration^74^. Similarly, BDNF serum levels are lower in early-stage PD patients compared to controls, whereas in later stages BDNF serum levels correlate positively with duration and disease severity^115^, possibly reflecting a compensatory mechanism. Targeted α-syn overexpression negatively impacts BDNF gene and protein expression, as well as downstream BDNF signaling^116–118^. Conversely, α-syn silencing results in an upregulation of BDNF mRNA^117^. Retrograde transport of BDNF is also impaired in neurons that overexpress α-syn^118^. Neurons with PFF-seeded α-syn inclusions have reduced retrograde transport of the BDNF receptor, TrkB^49^, and overexpression of α-syn has also been shown to inhibit BDNF-TrkB signaling in vitro^119^. Collectively, these studies suggest that pathological α-syn decreases levels of BDNF, interferes with retrograde BDNF transport, and decreases TrkB levels and BDNF-TrkB signaling. It is therefore possible that increased BDNF expression could counteract the pathological consequences of synucleinopathy.

Indeed, BDNF has been linked to positive effects on many of the same cellular processes impacted with LBs (Fig. 1). Specifically, BDNF increases mitochondrial oxidative efficiency and combats mitochondrial dysfunction^73,120^, enhances synaptic transmission^121^, and promotes synaptic plasticity^122^ including increasing dendritic spine density^123^. Further, BDNF improves presynaptic dopamine release and reuptake^114^ and can protect nigral dopamine neurons from neurotoxicant insult in mesencephalic dopamine neuron cultures and rodent and non-human primate models^124,125^. Beyond synucleinopathy, impaired BDNF signaling has been documented in other neurodegenerative disorders, including Alzheimer’s disease^126,127^. PD is a heterogeneous disorder with co-pathologies that can include amyloid, which may also be impacted by BDNF. In summary, BDNF-TrkB signaling has the potential to exert a multitude of disease-modifying effects on the basal ganglia and other nuclei.

The earliest studies examining whether increased BDNF levels can be neuroprotective reported positive effects in toxicant models of PD^124,125,128–130^. What remains unknown is whether therapeutic strategies that increase BDNF or TrkB signaling can exert neuroprotective effects within the context of synucleinopathy. In the α-syn A53T mutant mouse model, the FDA-approved drug, Gilenya (FTY720/fingolimod) decreased α-syn aggregation in the enteric nervous system and alleviated gut motility symptoms in a BDNF-TrkB dependent manner^131^. Specifically, the use of TrkB antagonist (ANA-12) in young A53T transgenic mice exacerbated constipation and increased synucleinopathy in the gut, both of which were mitigated by Gilenya treatment. Some investigations have explored exercise as a less invasive way to stimulate BDNF-TrkB, as exercise is known to increase BDNF production^132^. This approach has proven promising in several preclinical animal models of PD in which exercise-induced BDNF upregulation and significantly reduced α-syn aggregation were observed, with no change to soluble α-syn^133–136^. Another approach to increase endogenous production and release of BDNF is through high-frequency stimulation^101,137^. We have previously demonstrated that subthalamic nucleus deep brain stimulation (STN DBS) specifically elevates BDNF mRNA and protein throughout the basal ganglia^138–142^. Moreover, TrkB blockade prevented the neuroprotection normally associated with stimulation^143^. STN DBS increases striatal BDNF despite the presence of PFF-seeded α-syn inclusions, which partially restored the normal corticostriatal BDNF relationship^144^. Collectively, these data present a compelling argument for the potential of DBS-enhanced BDNF to mitigate nigrostriatal terminal dysfunction. Future studies will be required to determine whether DBS-mediated effects on BDNF translate into neuroprotection from α-syn inclusion-associated degeneration.

## Conclusion

Investigations using postmortem PD tissue and PFF-exposed cells have revealed multiple pathogenic processes associated with the presence of misfolded pathological α-syn inclusions. Further research is required to determine whether the insight gleaned from understanding LB-associated pathogenesis can be translated into therapies for disease modification in PD.

### Reporting summary

Further information on research design is available in the Nature Research Reporting Summary linked to this article.

## Supplementary information

Reporting Summary

## Data Availability

No datasets were generated or analyzed during the current study.
